# Estrogens as Antioxidant Modulators in Human Fertility

**DOI:** 10.1155/2013/607939

**Published:** 2013-11-20

**Authors:** A. Mancini, S. Raimondo, M. Persano, C. Di Segni, M. Cammarano, G. Gadotti, A. Silvestrini, A. Pontecorvi, E. Meucci

**Affiliations:** ^1^Division of Endocrinology, Department of Internal Medicine, Catholic University of the Sacred Heart, Rome, Italy; ^2^Institute of Biochemistry and Clinical Biochemistry, Catholic University of the Sacred Heart, Rome, Italy

## Abstract

Among treatments proposed for idiopathic male infertility, antiestrogens, like tamoxifen, play a possible role. On the other hand, oxidative stress is a mechanism well recognized for deleterious effects on spermatozoa function. After reviewing the literature on the effects of estrogens in modulation of antioxidant systems, in both sexes, and in different *in vivo* and *in vitro* models, we suggest, also on the basis of personal data, that a tamoxifen treatment could be active via an increase in seminal antioxidants.

## 1. Introduction

Idiopathic infertility is a condition that refers to those men who have abnormal semen parameters without an identifiable cause based on history, physical examination, and currently available laboratory and radiographic examination.

The fact that is impossible to determine the precise etiology of this form of infertility does not allow for making a rational treatment but only an empirical one; spermatogenesis is supported acting on various pathways. 

A wide number of agents have been proposed as specific treatment for men with infertility but there is no strong evidence of any therapy and nowadays there is still no consensus between various societies that are researching in this specific field.

## 2. Oxidative Stress

Oxidative stress (OS) is defined as the unbalance between production of free radicals, molecules characterized by high reactivity due to one or more unpaired electrons in the external orbital, and antioxidant defenses in the biological systems. Nowadays, it is widely accepted as an important pathogenetic mechanism in different diseases [1]. Among free radicals the most important and studied are reactive oxygen species (ROS), of which the most *in vivo* production occurs most of all during oxidative processes of energetic substrates in the mitochondrial respiratory chain [2, 3]. However, other important kinds of free radicals exist, besides ROS, among which nitrogen reactive species are the most studied [4]. An augmented ROS production can be the consequence of an augmented electronic flow in the respiratory chain, when it is activated from an augmented energetic demand or an augmented contribution of substrates [5], as what occurs in obesity. In leukocytes and many other cytotypes, as endothelial and mesangial cells, fibroblasts, thyrocytes, oocytes, Leydig cells, adipocytes, Epstein-Barr infected cells and neoplastic cells, ROS generation has been assessed to have a positive physiologic functional role, different from respiratory burst [6]. However an uncontrolled production of free radicals was linked to many pathologic events, as rheumatoid arthritis and myocardial infarction [2], and in general ROS damage occurs in inflamed tissues, characterized from cellular lysis and intracellular content release. Moreover in diabetes mellitus, oxidation, accompanying glycation *in vivo*, supports the formation of more permanent, irreversible chemical modifications on different kinds of molecules; metalcatalyzed autoxidation of glucose in the presence or absence of protein is paralleled by the generation of reactive oxygen species. The formation of glycoxidation products *in vivo* depends on the relative glucose concentrations, but also on the local oxidative environment. On the other hand in diabetic patients antioxidant capacity is decreased, finally resulting in not only increased susceptibility to oxidative stress [7].

It is possible to characterize different cellular defensive mechanisms against the free radical damage [2] which act in the endoplasmic network, mitochondria, plasmatic membrane, peroxisomes, and cytosol as well as extracellular ambient. The first mechanism is the prevention of production or the rapid inactivation of free radicals, due to the activity of several enzymes, like catalase, peroxidase glutathione complex, and superoxide dismutase (SOD), or of transition-metals binding proteins, like transferrin, ferritin and ceruloplasmin. The second mechanism determines an interruption of propagation of the lipid peroxidation chain by a reaction with the intermediate radicals and the consequent their neutralization. This mechanism is related to the role of molecules called “scavengers,” which can be water soluble, as albumin, bilirubin, ascorbic acid, urates and thiols, or liposoluble, as vitamin E and coenzyme Q_10_, the only liposoluble antioxidant synthesized in the living organisms. The mobility of scavengers, particularly the liposoluble ones, and above all at membrane level, allows for intercepting radicals and transformin them in more stable molecules and therefore stopping the damage chain. Sometimes scavengers can be regenerated. The third defensive mechanism uses processes which remove molecules damaged by oxidative attack, allowing the reconstitution of normal structures (e.g., specific phospholipases remove the peroxidized fatty acids, making possible the reacylation of damaged molecule by an acyl-CoA and the respective enzyme). Recently a lot of methods have been developed in order to measure the total antioxidant status in biological fluids. Total antioxidant capacity (TAC) is a measurement of the nonenzymatic antioxidants, that are primarily extracellular, as ascorbate, urates, albumin, tocopherol, and glutathion. They are “chain-breaking” molecules able to block the propagation chain of lipid peroxidation and to prevent the amplification of radical generation and the subsequent biochemical damage. Differently from enzymes, they are consumed at the moment in which they act and this fact could explain the reduction in their levels in biological fluids producing ROS (e.g., the high levels of ROS and a low TAC in the seminal fluid of infertile males suggests oxidative stress is associated with a variety of etiologies of male infertility) [8]. TAC is considered an index of the antioxidant status of a biological sample better than the measurement of one or more specific antioxidants, that could not carefully reflect the combined effect of the various antioxidants and their “collaboration” during the oxidative stress. We have applied the method of Rice-Evans and Miller [9], with some modifications [10], to different endocrine diseases and the results are summarized in the following paragraphs. It consists in TAC evaluation by using the system metmyoglobin-H_2_O_2_, as source of radicals, in presence of the chromogen 2,2^I^-azinobis-(3-ethylbenzothiazoline-6-sulphonate), the radical form of which is spectroscopically detectable after a latency time (LAG) due to antioxidants presence and therefore proportional to their content.

The involvement of OS in male infertility is well established [11], but studies on the relationships between sperm quality, seminal antioxidant, and OS continue to be published [12, 13]. An increase in seminal ROS is associated with sperm DNA fragmentation; the better parameter to identify such condition should be ROS-TAC score [14]. Recently it has been shown that not only seminal but also blood antioxidants can be considered biochemical markers to support sperm quality evaluation [15]. Different prooxidative conditions can negatively influence sperm viability and motility, sharing OS as common end, including exposure to radiation, extern temperature, drugs and toxins, heavy metals, smoking, biological hazards, and electromagnetic [16, 17]. 

Finally, new mechanisms have been recently discovered, such a ROS-induced uncoupling between electron transport and ATP synthesis, by evaluation of sperm mitochondrial respiratory activity with a polarographic assay in hypotonically treated sperm cells [18]; on the other hand, mitochondrial independent mechanisms, such as alteration of ATP utilization or contractile apparatus of the flagellum, have been hypothesized, since menadione (mitochondrial generator of superoxide) and hydrogen peroxide caused an immediate disruption of motility, with delayed or no decrease in ATP content, respectively, in a model of boar sperm [19].

Considering all the reported data, antioxidant therapies have a strong physiopathological rationale for the employment in idiopathic male infertility [20].

## 3. Estrogens and Oxidative Stress: *In Vitro* and *In Vivo* Studies

There is evidence that 17-*β*-E_2_ induces ROS production in mitochondria as signal-transducing messengers. In this way it activates the binding of three oxidant-sensitive transcription factors: AP-1, CREB, and nuclear respiratory factor 1 [21]. However, the production of ROS seems to exert negative effect and explain a sex-related differential longevity [22]. Estrogens bind to E-receptors and, via activation of MAP kinase and NF-*κ*B pathways, result in an upregulation of antioxidant enzymes. Moreover, the oxidative damage of mitochondrial DNA is fourfold higher in males than females. Findings in transgenic animal overexpressing Mn-SOD or catalase strongly support this view [23]. 

Estrogens exert protective effects in different cell types. E_2_ increases glutathione levels in different cellular CNS models, both neuronal and glial cells [24]. In other models (rat liver microsomes, with lipid peroxidation determined by measuring TBARS after exposure to various prooxidants) catechol estrogens appeared the most potent antioxidants, even if estrogens and catechol estrogens interact with the peroxidative process at different levels [25]. Protective effects of estrogens on ter-butylhydroperoxide-induced hepatocyte damage were studied *in vitro*, suggesting that this protection may be related not only to their antioxidant activity against free radicals but also to the maintenance of the normal redox status of cells, which partially restores intracellular GSH levels [26].

Neutrophil granulocytes play an important role in atherogenesis also through their free radical generation. Despite the conflicting results in the literature [27, 28], a suppressive effect on superoxide anion production of human neutrophil granulocytes by 17-*β*-estradiol, progesterone and testosterone was demonstrated [29]. 

Despite these effects, estrogen-like compounds mediate DNA damage by ROS generation in human lymphocytes and sperm [30].

In ovariectomized Wistar rats, administration of estradiol reduced MDA, together with a slight decline in catalase and SOD, with no modification of vitamins A and E [31]. The different effects of estrogens on the glandular and aglandular portions of endometrium were studied in ovariectomized ewes: treatment with E_2_ decreased SOD1, catalase and GPX in both the endometrial tissues, and GSR only in the glandular part. Progesterone did not influenced SOD2, catalase, GPX and GSR but induced a SOD1 decrease in the aglandular tissue [32].

As far as vascular system is concerned, estrogen depletion after ovariectomy in rats induced, via oxidative stress, the activation of heme-oxygenase 1 (HO-1), an inducible stress protein. NO/iNOS system contributes to the induction of HO-1, which may subsequently suppress iNOS activity. Estradiol replacement reversed these effects [33]. 

An interesting review on the cellular and molecular mechanisms underlying vasculoprotective action of estrogens [34] explains how E_2_ can act through a nongenomic stimulation of membrane/intracellular mediators and/or the classical genomic pathway. In this way E_2_ improves vasomotion by modulation of vasoconstrictor and vasodilator systems. It also affects remodeling of the vascular wall and modulates vascular inflammatory response. Recently, the effect of oxidative stress in female reproduction has been reviewed, and it appears to be involved in the pathophysiology of infertility and assisted fertility [35]. Oxidant status of the cell modulates angiogenesis, which is critical for follicular growth, corpus luteum formation, endometrial differentiation and embryonic growth [36]. 

Erythrocyte GSH-Px is influenced during the menstrual cycle: higher GSH-Px activity was found from the later follicular to early luteal phase. A significant correlation was observed between E_2_ and GSH-Px cycle-related changes, while no significant cycle phase-dependent variation was found in pyruvate kinase activity [37].

Estrogen treatment reduces peroxidation of neuronal synaptic membrane in postmenopausal women, thus preventing neurovascular and neurodegenerative disorders [38]. A large number of neurological and psychiatric diseases like Parkinson, SLA, dementia and schizophrenia show an enhanced ROS production and beneficial effects due to antioxidant properties of estrogen have been shown [39].

Even if these data indicate that E_2_ is a potent preventative agent against neurodegenerative diseases, by activating antioxidant defense systems, scavenging ROS, limiting mitochondrial protein damage, improving electron transport chain activity and reducing mitochondrial DNA damage, the high oxidative cellular environment in neurodegeneration makes E_2_ a poor agent for the treatment of a overt disease. Oxidative stress stimulates the production of the hydroperoxide-dependent hydroxylation of estradiol to the catechol estrogen metabolites, which can undergo ROS producing redox cycling, setting up a self-generating toxic cascade offsetting any antioxidant/antiapoptotic effects generated by the parent estradiol. Additional factors, related to the disease, can further complicate such a condition: for instance a dysregulation of the catecholamine system could alter COMT-catalyzed methylation, preventing removal of redox cycling catechol estrogens from the system enhancing prooxidant effects of E_2_ [40]. 

The above reported experimental data support the controversies on estrogen replacement therapy and cardioprotection [41]. Initially attributed to LDL lowering and HDL increasing, this protective effect is centered on decreased LDL oxidation. A direct effect on arterial tissue and a modulation of vascular reactivity through NO and prostaglandin synthesis, based on both receptorial and immediate nongenomic mechanisms, are also involved. Recently another mechanism was hypothesized implicating the esterification of estrogens in HDL and the transfer to LDL. Despite these mechanisms, two recent placebo-controlled studies on women with CHD failed to show beneficial effects of hormone replacement therapy on coronary events, even if mutations in thrombogenic genes may represent an important confounding factor. 

Postmenopausal women develop visceral obesity and insulin resistance and are at increased risk for type 2 diabetes [42]. In the management of menopausal disturbances both natural steroid and Selective Estrogen Receptor Modulators (SERMs) could be useful for antioxidant activity [43], but both positive and negative effects have to be considered when evaluating the role of estrogen in cardiovascular disease [44, 45].

## 4. Treatment for Infertile Men with Idiopathic Oligo-/Azoospermia with Tamoxifen

Selective Estrogen Receptor Modulators have been proposed among the most promising therapies. These drugs differ from pure receptor agonists and antagonists because their action is different in various tissues, granting the possibility to selectively inhibit or stimulate estrogen-like action in various tissues. Both clomiphene citrate and tamoxifen (TMX) have been suggested as empiric treatments for male infertility, with different reports in the last decades. TMX has reached a leading role as a suitable gonadotropin stimulator rising directly and indirectly the testicular function with a clear advantage on clomiphene that, according to its estrogenic effect, would seem to lower the sex-hormone binding protein and testosterone levels depressing spermatogenesis [46–50].

The effects of different SERMs on hypothalamic-pituitary-testicular axis have been investigated in men with idiopathic oligozoospermia: TMX (20 mg/daily), toremifene (60 mg/daily) and raloxifene (60 mg/daily), administration resulted in a significant increase in gonadotropin levels, which was more marked for TMX and toremifene compared with raloxifene [51].

Several medical trials assessed the efficacy of TMX in infertility treatment, but the results were not strong enough to justify general acceptance [52]. Similarly, a multicentric WHO study showed only an 8% increase in pregnancy rate in couples receiving clomiphene versus placebo [53]. Another meta-analysis of antiestrogen therapy (clomiphene and tamoxifen), including randomized and placebo-controlled studies, concluded that such a treatment had no significant influence on pregnancy rate [54]. Again, the question has been reevaluated in recent years. Another meta-analysis, which excluded some previously considered studies (in which vitamin C was used as placebo) and included other randomized controlled trials, showed an overall beneficial effect on pregnancy outcome (pooled OR: 2.42, 95% CI 1.48, 3.94 *P* = 0.00004) [55]. A modest but significant increase in sperm concentrations and a statistically significant improvement in sperm motility were shown in the same study.

TMX seems to have a strong effect on sperm count and concentration in eugonadal patients but it does not improve other semen values such as volume, pH, motility, morphology, and viability and this could be related to its effect on seminiferous tubules where it should ameliorate first steps of spermatogenesis [56]. A recent article suggests a better effect of TMX in patients with low FSH levels before starting the therapy [57] suggesting the need for a well-functioning hypothalamic-pituitary-gonadal axis.

The TMX therapy has been supplemented with low dose of Testosterone Undecanoate (TU), a weak androgen that in this context does not interact with the axis [56] but improves other sperm parameters, except for sperm count, and the chances of conception.

The efficacy of TMX association with coenzyme Q10 has been investigated in a group of 183 patients affected by idiopathic oligoasthenozoospermia, randomly assigned to different treatment schedules: TMX + CoQ10, TMX, or CoQ10 alone. In the first two groups, as expected, FSH, LH, and testosterone levels significantly increased; sperm motility and % of morphologically normal forms were significantly higher in TMX + CoQ10 and CoQ10 groups and slightly increased, but without statistical significance, in the TMX group [58].

Again, all above reported studies refer to SERM as “empiric” therapy. However, due to the role of ROS in male infertility and the role of estrogens in modulating antioxidant systems, as reviewed in the previous paragraph, they could also have a pathophysiological basis to explain their positive effects.

## 5. Antiestrogens and Total Antioxidant Capacity

No studies are reported on the effects of TMX on antioxidant capacity in seminal plasma. The role of antioxidants in male infertility treatment is well recognized [59, 60]; other therapies can influence TAC suggesting the possibility that hormonal therapy, at least in part, has beneficial effects through the balance between ROS and antioxidants. We previously reported [61] that a treatment with CoQ10 is able to increase seminal plasma TAC, also in low dose schedule (100 mg/daily), slightly influencing seminal parameters of varicocele patients. On the other hand, a FSH treatment (225 UI/week for three months) induced a trend toward increase in endogenous seminal plasma CoQ10 levels in oligoasthenozoospermic subjects [62].

Recently we have investigated the effects of TMX administration (20 mg daily for a three-month period) in a group of 5 patients, aged 27–32 years, affected by idiopathic infertility. Criteria of exclusion were varicocele, primary or secondary hypogonadism, and low urinary tract inflammation. They were oligospermic, with a mean basal sperm concentrations of 14.7 ± 10.3 × 10^6^/mL, 32.8 ± 22.3% of progressive motile cells, and 30.0 ± 7.8% normal morphology. LAG values were significantly increased by hormonal treatment (120.0 ± 14.1 versus 102.5 ± 10.6 sec). 

These very preliminary observations need to be validated in a large sample and in double-blind, placebo-controlled study. However they again suggest the possible role of estrogens as modulators of antioxidants system in human semen and furnish a further rationale, on biochemical basis, for antiestrogens in male infertility.
